# Effects of Intermittent Fasting on Specific Exercise Performance Outcomes: A Systematic Review Including Meta-Analysis

**DOI:** 10.3390/nu12051390

**Published:** 2020-05-12

**Authors:** Joana M. Correia, Inês Santos, Pedro Pezarat-Correia, Cláudia Minderico, Goncalo V. Mendonca

**Affiliations:** 1 Neuromuscular Research Lab, Faculdade de Motricidade Humana, Universidade de Lisboa, Estrada da Costa, 1499-002 Cruz Quebrada, Dafundo, Portugal; joanacorreia-19@live.com.pt (J.M.C.); ppezarat@fmh.ulisboa.pt (P.P.-C.); 2 CIPER, Faculdade de Motricidade Humana, Universidade de Lisboa, Estrada da Costa, 1499-002 Cruz Quebrada, Dafundo, Portugal; isantos@fmh.ulisboa.pt (I.S.); cminderico@gmail.com (C.M.); 3 Laboratório de Nutrição, Faculdade de Medicina, Universidade de Lisboa, 1649-028 Lisboa, Portugal

**Keywords:** nutrition, aerobic capacity, anaerobic capacity, performance, training, muscle strength

## Abstract

Intermittent fasting (IF) has been studied in athletes during Ramadan and in those willing to decrease adiposity while maintaining or increasing lean body mass. The purpose of this systematic review was to summarize the effects of IF on performance outcomes. We searched peer-reviewed articles in the following databases: PubMed, Web of Science and Sport Discus (up to December 2019). Studies were selected if they included samples of adults (≥18 years), had an experimental or observational design, investigated IF (Ramadan and time-restricted feeding (TRF)), and included performance outcomes. Meta-analytical procedures were conducted when feasible. Twenty-eight articles met the eligibility criteria. Findings indicated that maximum oxygen uptake is significantly enhanced with TRF protocols (SMD = 1.32, *p* = 0.001), but reduced with Ramadan intermittent fasting (Ramadan IF; SMD = −2.20, *p* < 0.001). Additional effects of IF may be observed in body composition (body mass and fat mass). Non-significant effects were observed for muscle strength and anaerobic capacity. While Ramadan IF may lead to impairments in aerobic capacity, TRF may be effective for improving it. As there are few studies per performance outcome, more research is needed to move the field forward.

## 1. Introduction

Caloric restriction (CR) and intermittent fasting (IF)—i.e., periods of voluntary abstinence from food and fluid intake—are methods of energy deprivation [1]. IF is a term that encompasses many definitions involving fasting for varying periods (usually for 12 h or more). It also implicates a limited feeding time-window, with or without CR. One example is time-restricted feeding (TRF), or fasting for multiples days or weeks (i.e., alternate-day fasting or whole day fasting) [2,3]. IF is a key component used in many religious practices and has mostly been studied in athletes during Ramadan [4,5] and in those willing to decrease adiposity while maintaining or increasing lean body mass [6,7]. Ramadan is the major religious period of the Islamic calendar and is a fundamental rule of Islam, where Muslims abstain from ingesting food and liquids between sunrise and sunset during a month-long period [8]. Even though Muslims eat ad libitum after sunset (Iftar meal) and before dawn (Sahur meal), CR (either with or without exclusion of specific types of foods) is often a key feature of Ramadan IF [9]. This IF approach may negatively affect various aspects of human health and exercise performance, such as psychological factors (e.g., motivation), physiological mechanisms (e.g., muscular activation) and biochemistry (e.g., plasma volume and glycogen stores) [5,10,11]. However, according to the available data, short-term fasting (7 days) does not adversely impact aerobic performance [5,12], walking efficiency [13] nor maximum oxygen uptake (VO_2max_) [8,14,15,16]. In contrast, longer fasting periods may exert a deleterious effect on VO_2max_ as well as on the inotropic response to exercise and submaximal oxygen kinetics [9,13,17,18,19]. It is believed that the etiology of poor exercise performance during Ramadan involves the negative energy balance induced by a reduction in energy intake, altered circadian rhythm, sleep deprivation, heightened psychological stress, low levels of blood sugar and dehydration (that inevitably accompany this specific IF regimen) [17,18,20,21,22].

Controlled studies investigating the effectiveness of TRF (vs. constant daily CR) in decreasing adiposity, while maintaining muscle mass and strength, are scarce and conflicting. Schoenfeld contended that exercise while fasting (e.g., after an overnight fast) is not more effective in reducing adiposity than exercising in a fed state, and may possibly be detrimental for muscle and strength gains due to the potentially increased proteolysis [23]. However, three recent randomized controlled trials [2,6,24], including participants undergoing 8 weeks of resistance training combined with either a normal diet or a TRF protocol, indicate that this approach may be beneficial for improving body composition (i.e., preserved fat free mass and reduced fat mass) while exerting no detrimental effects on muscle strength. While Moro et al. found no differences between this IF regimen vs. a control diet on increasing leg-press one repetition maximum [6], Tinsley et al. observed that TRF resulted in greater enhancements inlower-body muscle strength and endurance, as well as in upper-body muscle endurance [2].

There is some evidence that, during the fasting periods, TRF predisposes to a reduced insulin-induced inhibition of adipose tissue lipolysis. This is supported by past research showing that plasma free fatty acid and β-hydroxybutyrate concentrations are enhanced after overnight fast in healthy young men [25]. Interestingly, these effects are accompanied by an improved rate of insulin-mediated whole body glucose uptake after 15 days of TRF [25]. Thus, this IF approach seems compatible with a more favorable metabolic profile and this likely contributes to positive variations in body composition (maintenance of fat free mass and reductions in fat mass) and exercise performance. Yet, there is a paucity of data on the effects of different IF protocols on specific parameters of physical performance. Specifically, there are no systematic and meta-analytic data on the effects of Ramadan IF vs. TRF on specific outcomes of performance responses to exercise. Therefore, this systematic review including meta-analysis aimed at summarizing the current evidence on the interaction between Ramadan IF vs. TRF and specific parameters of physical performance, namely VO_2max_, vertical jump height, 30 min running distance, Wingate mean and peak power output. The effects of these two approaches of IF on body composition adaptations, namely, absolute body mass and relative fat mass, were also synthesized to explore if these may partially explain the effects (or lack thereof) on exercise performance. We hypothesized that IF might contribute for enhancing physical performance as well as body composition.

## 2. Methods

This systematic review including meta-analyses was conducted according to the PRISMA statement (Preferred Report Items for Systematic Reviews and Meta-analysis) [26]. The methodological aspects were previously specified and registered in a protocol (PROSPERO registration number: CRD42019126847).

### 2.1. Eligibility Criteria

Articles published between 1980 and 2019 investigating the relationship between IF and physical outcomes were retrieved. Studies were selected if they: (i) included samples of adults (≥18 years); (ii) had an experimental or observational design; (iii) investigated Ramadan IF, time-restricted feeding, time-restricted eating and fasted state; and (iv) included performance outcomes. Studies with clinical outcomes, without physical parameters, with non-human samples, reviews, case studies, as well as published abstracts were not included.

### 2.2. Search Strategy

Searches of peer-reviewed articles published until December 2019 were conducted in three databases: PubMed, Web of Science and Sport Discus. Searches included various combinations of: (i) terms concerning the nutritional intervention, namely IF, time-restricted feeding, time-restricted eating and fasted state; and (ii) terms reflecting the outcomes of interest (i.e., exercise, muscle strength, anaerobic capacity, aerobic capacity and performance). Supplementary searches, including manual searches of previous key reviews (e.g., [14]) and reference lists of key retrieved articles were conducted.

### 2.3. Screening and Data Extraction

Three authors independently screened all titles and abstracts of the studies that emerged in the literature search for potential inclusion eligibility. Duplicate entries were removed. Information about each article (i.e., authors and year of publication), participants (i.e., sample size and demographics), study design, intervention characteristics (i.e., methods, protocols and length of intervention), and outcomes of interest (primary: aerobic changes, anaerobic changes, and muscle strength changes; secondary: body composition changes) was extracted. Uncertainties were resolved by consensus.

### 2.4. Quality Assessment

The Quality Assessment Tool for Quantitative Studies developed by the Effective Public Health Practice Project (EPHPP) was used to assess study quality [27], evaluating five key methodological domains: selection bias, study design, confounders, data collection method and withdrawals/dropouts. Since it is not possible to blind participants regarding a fasting state, the sixth item of the original scale (blinding) was not assessed. For each study, the methodological quality of each domain was classified as strong, moderate and weak. A global rating was calculated based on the scores of each component. The five domains and overall quality were rated independently by two authors, and differences were discussed with a third author to reach a consensus. Inter-rater agreement across categories varied from moderate (Cogen’s k = 0.429) to strong (k = 0.739).

### 2.5. Data Synthesis

For each study, results were summarised by: (i) intervention characteristics (intervention methods, exercise or test protocols and length of each trial); (ii) changes in aerobic capacity; (iii) changes in anaerobic capacity; (iv) changes in muscle strength; and (v) changes in body composition.

### 2.6. Data Analysis

The Comprehensive Meta-Analysis (CMA) Software version 3.0 (Biostat, Inc., Englewood, NJ, USA) was used to conduct meta-analyses [28]. We performed separate meta-analyses for each outcome (VO_2max_, Wingate mean power output, Wingate peak power output, vertical jump height, body mass and fat mass). Meta-analyses were performed using fixed-effects models, in which the summary effect size (ES) is the standardized mean difference (SMD) of a distribution [29,30]. Fixed-effects models were chosen because most analyses included a limited number of studies (k < 6) [29]. The only exceptions were some of the analyses for one of the secondary outcomes, body mass, in which a random-effects model was used given the higher number of studies (k ≥ 6). SMD were calculated based on sample size, standard differences in means (between pre- and post-intervention time points and, whenever possible, between pre-post data of both intervention and control groups) and effect direction, and were interpreted according to Cohen’s specifications, with values of 0.2, 0.5 and 0.8 for small, medium and large SMD, respectively [31]. The 95% confidence interval (CI) and corresponding *p* values were considered as indicators of statistical significance.

Heterogeneity was estimated to evaluate the amount of variation in the effects of the included studies. This was done using the following approach: (i) the Cochran’s Q statistic [32], for which a significant *p* value (<0.05) demonstrates that there is heterogeneity in the SMD between studies; and (ii) the *I*^2^ statistic [33] that assesses the proportion of observed variation in SMD between studies that is due to real variability and not influenced by low statistical power. The *I*^2^ ranges from 0% to 100%, where a value of 0% specifies no observed heterogeneity, 25% reflects low, 50% moderate and 75% high heterogeneity [33]. To explore if the results were affected by different fasting regimens (i.e., Ramadan IF vs. TRF) we performed sensitivity analyses. Some studies did not provide sufficient data to estimate the SMD and therefore were not included in the meta-analyses.

## 3. Results

### 3.1. Study Selection

**Figure 1:**
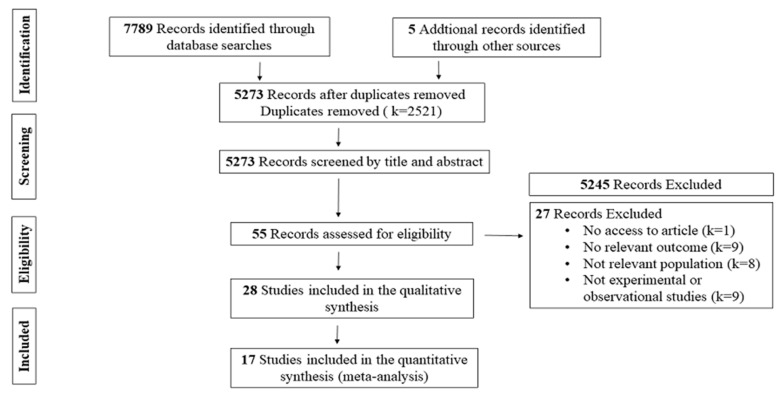
Flow chart of the methodology for the search results.

PubMed, Sport Discus and Web of Science searches yielded 7789 publications. To avoid missing potentially relevant studies, five articles were added through manual searches from previous key reviews and reference lists of key retrieved publications. After duplicates removal (k = 2521), 5273 articles were assessed for eligibility; of these, 5245 were excluded based on title and abstract screening, leaving 55 potentially relevant. Twenty-eight articles met all eligibility criteria and were included in the present review (Figure 1).

### 3.2. Study Characteristics

The characteristics of all included studies are summarised in Table 1. Half of the studies were non-controlled trials (k = 14), mainly aiming at performance outcomes (aerobic, anaerobic and muscle strength adaptations) and changes in body composition. The majority of IF protocols were conducted in laboratories (k = 23) and in gymnasiums or training camps (k = 6). More than half were undertaken during Ramadan (k = 15) and the others explored the effects of TRF vs. normal diet during a period of 8 weeks (k = 3), 6 weeks (k = 3), overnight fast (k = 1), and 3 to 10 days (k = 3). Participants performed five testing sessions during 8 h of fasting in one study. Sample sizes varied between 8 and 85 experimental participants, with the most common sample size corresponding to 10–20 individuals (k = 18). Participants’ age ranged between 18 and 39 years. Three studies included women, 14 studies enrolled healthy and moderately active samples, six studies tested sedentary or untrained men, and 10 focused on the responses of trained men (soccer players, running athletes or resistance-trained men). Seven studies focused on changes in anaerobic capacity, 15 in aerobic capacity, 12 in muscle strength and 21 in body composition. 

### 3.3. Quality Assessment

Table 2 shows the detailed classification of each quality domain and the overall methodological quality of each study. The methodological quality of the 28 eligible studies was rated as “strong”, “moderate” and “weak” in 4, 13 and 11 reports, respectively. Only six studies were randomized controlled trials and only two designs included a sample of individuals somewhat likely to be representative of the wider Muslim population experiencing IF. Therefore, 13 and 21 were rated as weak regarding the quality of study design and regarding selection bias, respectively. With regard to the adjustment of analyses for confounders, all studies (k = 28) were classified as strong because there were no bias arising from lack of controlled elements. In what concerns to reporting of withdrawals and dropouts, the 28 studies were rated as strong. Regarding data collection methods, all studies were rated as strong because they all used valid and reliable tools. 

### 3.4. Effects of Intermittent Fasting on Specific Outcomes

**Figure 2:**
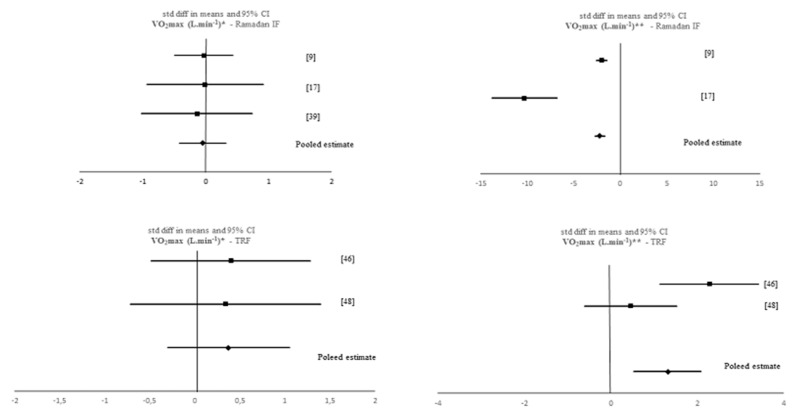
Forest plot of the effects from a fixed-effects meta-analysis shown as standardized mean difference with 95% confidence intervals on maximum oxygen uptake (VO_2max_) only Ramadan IF studies and only TRF studies. For each study, the square represents the standardized mean difference between pre- and post-intervention time points and, whenever possible, between pre- post-data of both intervention and control groups with the horizontal line intersecting it as the lower and upper limits of the 95% confidence interval. The rhombi represents the pooled estimated standardized mean difference. Note. * Pre-post tests, IF intervention; ** Pre-post tests, IF intervention vs. control.

**Figure 3:**
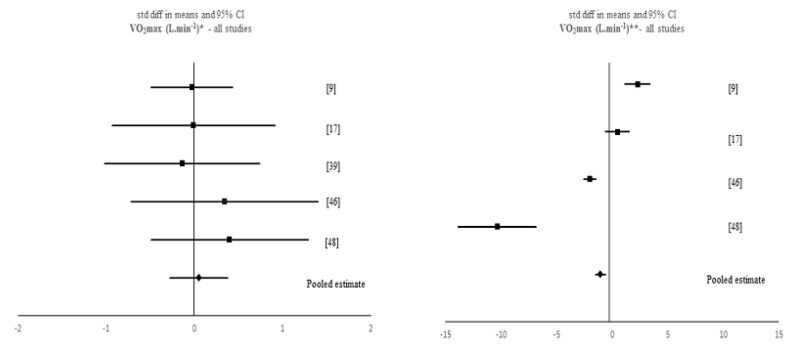
Forest plot of the effects from a fixed-effects meta-analysis shown as standardized mean difference with 95% confidence intervals on maximum oxygen uptake (VO_2max_) for all studies. For each study, the square represents the standardized mean difference between pre- and post-intervention time points and, whenever possible, between pre- post-data of both intervention and control groups with the horizontal line intersecting it as the lower and upper limits of the 95% confidence interval. The rhombi represent the pooled estimated standardized mean difference. Note. * Pre-post tests, IF intervention; ** Pre-post tests, IF intervention vs. control.

**Figure 4:**
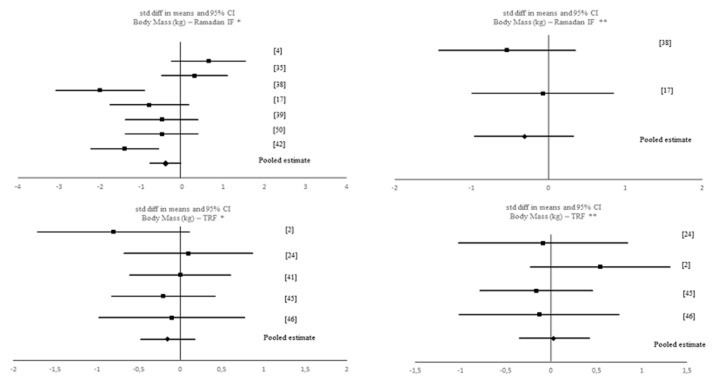
Forest plot of the effects from a random-effects meta-analysis shown as standardized mean difference with 95% confidence intervals on body mass for * Ramadan IF studies and forest plot of the effects from a fixed-effects meta-analysis shown as standardized mean difference with 95% confidence intervals on body mass for TRF studies. For each study, the square represents the standardized mean difference between pre- and post-intervention time points and, whenever possible, between pre-post data of both intervention and control groups with the horizontal line intersecting it as the lower and upper limits of the 95% confidence interval. The rhombi represents the pooled estimated standardized mean difference. Note. * Pre-post tests, IF intervention; ** Pre-post tests, IF intervention vs. control.

**Figure 5:**
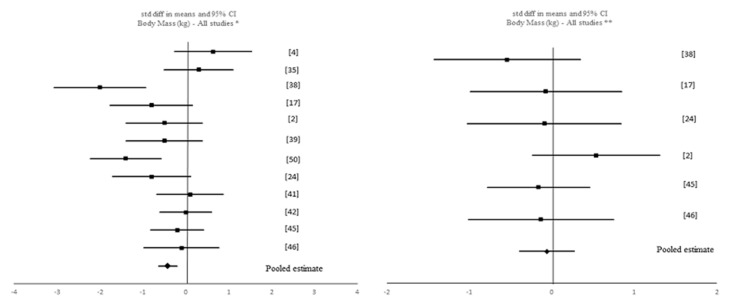
Forest plot of the effects from a random-effects meta-analysis shown as standardized mean difference with 95% confidence intervals on body mass for all studies. For each study, the square represents the standardized mean difference between pre- and post-intervention time points and, whenever possible, between pre-post data of both intervention and control groups with the horizontal line intersecting it as the lower and upper limits of the 95% confidence interval. The rhombi represents the pooled estimated standardized mean difference. Note. * Pre-post tests, IF intervention; ** Pre-post tests, IF intervention vs. control.

**Figure 6:**
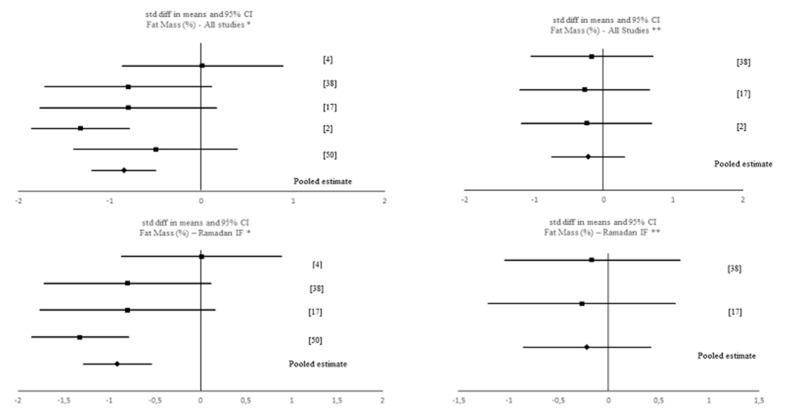
Forest plot of the effects from a fixed-effects meta-analysis shown as standardized mean difference with 95% confidence intervals on relative fat mass for all studies and for only Ramadan IF studies. For each study, the square represents the standardized mean difference between pre- and post-intervention time points and, whenever possible, between pre-post data of both intervention and control groups with the horizontal line intersecting it as the lower and upper limits of the 95% confidence interval. The rhombi represents the pooled estimated standardized mean difference. Note. * Pre-post tests, IF intervention; ** Pre-post tests, IF intervention vs. control.

A data analytic synthesis of the interaction between the four tested outcomes and IF is shown in Table 1. Table 3 and Table 4 show the meta-analytic results for the pooled estimates of these primary (i.e., muscle strength, aerobic capacity and anaerobic capacity) and secondary (i.e., body composition) outcomes, respectively, and Figure 2, Figure 3, Figure 4, Figure 5 and Figure 6 the respective forest plots. For muscle strength, we tested the results derived from the vertical jump. For aerobic capacity, we tested the results on VO_2max_ and distance covered during 30 min of running. For anaerobic capacity, we tested the results derived from Wingate testing (mean power and peak power). For body composition, we tested the results observed on body mass and absolute as well as relative fat mass.

#### 3.4.1. Primary Outcomes

##### Changes in Muscle Strength

Twelve studies presented data for the association between IF and muscle strength, with approximately 18% of the studies observing significant changes. Some of these studies reported satisfactory results as small increases in vertical jump height (k = 1) with Ramadan IF, and in upper- and lower-body (k = 1) or in upper-arm girth (k = 1) with TRF. In contrast, other studies reported significant decreases in maximal muscle power (k = 1) and in maximal voluntary contraction (k = 2) after Ramadan IF. Seven studies reported no significant changes in muscle strength when comparing pre- to post-IF time points.

A non-significant SMD for vertical jump height (k = 3; SMD = 0.012, 95% CI [-0.324, 0.348], *p* = 0.945; *Q* = 43.598, *p* < 0.001; *I*^2^ = 95%) was observed when meta-analysing pre-post data of Ramadan IF interventions. This pooled estimate remained non-significant when comparing pre-post data between the Ramadan IF interventions and the control groups.

##### Changes in Aerobic Capacity

Fifteen studies reported adaptations in aerobic capacity, with approximately 36% of them attaining significant changes in this specific outcome. Some studies obtained significant decreases in VO_2max_ (k = 1), heart rate (k = 1), and VCO_2_ (k = 1) after Ramadan IF, and performance (reduction of sprint performance, running speed or physical activity; k = 4) with both Ramadan IF and TRF protocols. Only one report showed significant increases in VO_2max_ and improved 3000 m running times. Approximately 18% of the studies (k = 5) showed unaltered values for VO_2max_, maximal heart rate and running speed post-Ramadan IF (k = 3) or post-TRF (k = 2).

Non-significant effects of IF on VO_2max_ were found when meta-analysing pre-post data of all IF interventions (*p* > 0.05). However, when comparing pre-post data between the IF interventions and the control groups, a significant, negative SMD was found when considering all studies (k = 4; SMD = −1.045, 95% CI [−1.488, −0.602], *p* < 0.001) and Ramadan IF studies only (k = 2; SMD = −2.204, 95% CI [−2.745, −1.663], *p* < 0.001). Moreover, there was evidence of high heterogeneity between all studies (*Q* = 80.05, *p* < 0.001, *I*^2^ = 96%) and between Ramadan IF studies (*Q* = 21.229, *p* < 0.001, *I*^2^ = 95%). Conversely, we obtained a significant positive SMD for VO_2max_ when considering TRF studies only (SMD = 1.315, 95% CI [0.543, 2.087], *p* = 0.001) and there was evidence of high heterogeneity between studies (*Q* = 5.323, *p* = 0.021, *I*^2^ = 81%). Concerning the effects of Ramadan IF on 30 min running distance, when comparing means between the Ramadan IF interventions and the control groups, non-significant results emerged (*p* > 0.05).

##### Changes in Anaerobic Capacity

Only seven studies (of 28) reported results for anaerobic changes, with approximately 11% of them showing negative but significant effects of IF on maximal speed during high-intensity effort and in response to the Wingate protocol after Ramadan IF (k = 1) and TRF (k = 2). Approximately 7% of the studies showed no significant impact of Ramadan IF on anaerobic exercise performance.

Non-significant effects for Wingate mean power were observed when meta-analysing pre-post data of all IF interventions, and Ramadan IF interventions only (*p* > 0.05). Regarding Wingate peak power, the pooled estimate including pre-post data of all IF interventions was marginally significant (k = 3; SMD = 0.758 (95% CI [−0.019, 1.535], *p* = 0.056; *Q* = 113.389, *p* < 0.001; *I*^2^ = 98%).

#### 3.4.2. Secondary Outcome

##### Changes in Body Composition

Body composition was frequently studied (k = 21), with 14 studies expressing it in absolute terms (kg) and seven studies expressing it in terms of relative values (%). Approximately 68% of the studies reported similar results when determining the impact of IF on weight loss. However, these changes were not statistically significant. Data on the association between IF and body composition were available with significant results for approximately 7% of the studies, reporting decreases in both relative fat mass and body mass (kg) (k = 1) or only in body mass (kg) (k = 1).

Meta-analytic results showed a negative, small but significant effect of IF on body mass when including all IF interventions (k = 12; SMD = −0.435 (95% CI [−0.811, −0.059], *p* = 0.023; *Q* = 30.718, *p* = 0.001; *I*^2^ = 64%). This pooled estimate remained significant with Ramadan IF interventions only (k = 7): SMD = −0.638 (95% CI [−1.248, −0.029], *p* = 0.04; *Q* = 23.256, *p* = 0.001; *I*^2^ = 74%); but not with TRF interventions only (k = 5): SMD = −0.152 (95% CI [−0.476, 0.173], *p* = 0.359; *Q* = 2.635, *p* = 0.621; *I*^2^ = 0%). When comparing pre-post data between the IF interventions and the control groups, no significant pooled estimates were observed (*p* > 0.05).

Concerning the results of the meta-analysis for relative fat mass including all IF interventions (k = 5), a pooled estimate of −0.848 was obtained (95% CI [−1.193, −0.502], *p* < 0.001; *Q* = 7.307, *p* = 0.121; *I*^2^ = 45%), therefore showing a negative significant effect of IF on relative fat mass. This pooled estimate remained significant when removing the only study with a TRF intervention: SMD = −0.910 (95% CI [−1.284, −0.535], *p* < 0.001; *Q* = 6.616, *p* = 0.085; *I*^2^ = 55%). When comparing pre-post data between the IF interventions and the control groups, no significant pooled estimates emerged (*p* > 0.05).

## 4. Discussion

The aim of this comprehensive systematic review including meta-analysis was to summarize the effects of two approaches of IF (Ramadan IF vs. TRF) on performance outcomes through adaptations in muscle strength, aerobic and anaerobic capacity, as well as in body composition. Twenty-eight studies were included with meta-analytic results suggesting that IF affects exercise performance, specifically VO_2max_.

During Ramadan IF, aerobic capacity was shown to be affected with a significant decrease in VO_2max_. One possible explanation for these findings is that the dehydration accompanying Ramadan might reduce blood volume, maximal cardiac output and muscle glycogen stores, thus affecting VO_2max_ [9]. However, it is important to reinforce that statistically significant decrements in aerobic capacity have only been verified in the early phases of Ramadan IF and that these reductions are normally small and tend to diminish during the second half of Ramadan [36]. Contrary to that seen with Ramadan IF, according to our findings, TRF exerts a positive impact on VO_2max_. A possible explanation for the improvements in VO_2max_ with TRF includes an increase of cardiac output due to an increase of sympathetic stimulation during exercise or a greater oxidative capacity in fat adapted muscle. Ultimately, the combination of these phenomena may lead to improvements in aerobic performance in euhydrated conditions [44,48]. Thus, training in the fasted state could provide an adjuvant stimulus to enhance training adaptations while preserving plasma glucose concentrations at normal levels during exercise [45,46].

Improvements in VO_2max_ following TRF may also be partially related to positive adaptations in body composition. Despite not being a universal finding, TRF has been shown to induce slight increases in fat free mass (e.g., [53]). Moreover, fasting triggers numerous hormonal adaptations culminating in enhanced metabolic rate and preserved lean mass (e.g., increased serum levels of noradrenaline and growth hormone) [46]. In addition, exercising while fasting increases the expression of sirtuin 1 (SIRT1) and phosphorylated AMP- activated protein kinase (AMPK), which have numerous effects on gene expression (i.e., regulation of mitochondrial biogenesis) [54]. This is important because there is undisputed evidence that, to improve athletic performance, it is fundamental to achieve an optimal balance between lean and body fat mass [54]. Nevertheless, some authors argue that it is necessary to pay attention to the general protein content that is ingested during feeding hours, as well as the final energy balance achieved on a daily basis [3,22]. Ramadan fasting, perhaps due to the socioeconomic circumstances and cultural differences between Muslim countries, is frequently associated with daily restriction in energy and fluid intake. Ultimately, this may contribute to the differences between TRF and Ramadan IF interventions on improving VO_2max_ results [55]. Nevertheless, it is important to note that some authors observed no Ramadan-associated changes in energy intake or in body composition [4].

Ramadan IF is also well known to enhance the individual capacity to oxidize fat [56,57]. This positive metabolic adaptation to Ramadan IF seems to be in agreement with our meta-analytic findings on body mass and fat mass. In fact, systematic reviews and meta-analyses specifically designed to test the effect of IF on body composition have consistently shown that IF represents an effective intervention for weight loss [58,59]. One possible explanation is that IF-associated weight loss results from reductions in fat mass that may be secondary to a state of enhanced-lipid utilization during exercise [6,25,60]. However, after considering the control group, our meta-analytic results for these parameters were non-significant. Importantly, no major changes in body composition were seen in cases where IF had non-detrimental effects on exercise performance [24,45]. Additionally, alterations in body mass are largely related to individual circumstances and may vary according to cultural differences and socioeconomic factors, as well as narrow family oriented and daily habits [21].

Our analyses showed non-significant effects of both Ramadan IF and TRF on anaerobic performance and muscle strength. Yet, the available literature on this specific matter has provided mixed results. For example, a significant reduction in sprint performance during the initial stages of IF has been reported in past research (i.e., first 2–3 days) [41,43]. One possible explanation for such decrement in anaerobic performance might be that short-term IF depletes muscle energy stores (e.g., reduced rate of anaerobic adenosine triphosphate synthesis) [36]. However, it has also been suggested that decreased sprint performance may be secondary to central, rather than peripheral fatigue [42]. In addition, it could be a consequence of altered perception of fatigue during exercise in the fasted condition. This might lead to a decrease in central drive compared to that seen in non-fasting conditions, thus reducing the capability of the central nervous system to maximally recruit all the available motor units during sprinting [42]. However, Karli et al. observed significant improvements in Wingate test performance, thus suggesting that this might not be the case [4]. Moreover, Aloui et al. showed that training in the fasted state (regardless of the time of day) induces significant improvements in the total distance covered during the yo-yo intermittent recovery test after Ramadan IF [51]. Controversially, Souissi et al. showed that, while Wingate performance (peak and mean power output) was not affected when tested in the morning during Ramadan IF, this was not the case for testing sessions scheduled for the afternoon/evening period (7 p.m. and 9 p.m.) [35]. Under these circumstances, there was a substantial reduction in anaerobic performance vs. the control period. Therefore, it seems that the effect of IF on anaerobic performance is modulated by time of day and that small changes in anaerobic performance with IF may reflect a state of altered perceived exertion, slowed reaction time or impaired muscle function (caused by dehydration paired by muscle glycogen depletion) [35,39,41].

Although past research showed that IF does not negatively affect vertical jump height or muscle damage, other reports observed small decreases in muscle strength [8,9,11,37,38]. It was argued that such negative effects arised from slight reductions in fat free mass, dehydration or even from alterations in energy metabolism, muscle buffering capacity during muscular contractions and different lengths of training performance [51]. Finally, another study revealed that Ramadan IF is effective for improving vertical jump height during the afternoon, but not during the morning period. This provides preliminary evidence that the relationship between IF and muscle strength might interact with the well-known circadian variation in sports performance [61]. Nevertheless, due to the specificities of Ramadan IF (e.g., eating and drinking abstinence from sunrise to sunset), these findings may not be generalizable to other IF protocols. For example, in most studies involving TRF protocols, the levels of macronutrient intake, training load, sleep quality and hydration were usually well maintained over time [2,6,24,40]. In contrast, research designs focusing on Ramadan IF reported an overall reduction in carbohydrate intake, and this is compatible with a depletion of muscle glycogen stores [62]. The limited availability of carbohydrates within the muscle tissue impairs physical performance during high-intensity exercise [63]. Nevertheless, there is compelling evidence that the ingestion of selected amino acids or their metabolites during IF may be beneficial for lean mass maintenance or accretion [24]. In particular, it has been argued that leucine, a branched-chain amino acid, plays a pivotal role in stimulating protein synthesis through the mTOR signalling cascade, thus modulating these physiological effects [24,64]. Unfortunately, due to the unavoidable mild dehydration experienced during the hours of Ramadan IF, metabolic heterostasis is further exacerbated by this religious practice, and this impacts exercise performance negatively [34,62,65]. Finally, the effects of Ramadan IF on the physiological responses to exercise and training are inseparable from its long-lasting implications on sleep schedule and psychological, as well as social habits (a combination of factors that exert a negative influence on exercise performance) [36,66].

The present review has several limitations that need to be addressed. First, the number of studies per outcome included in the meta-analyses was small, thus reducing statistical power. The lack of a comparison group in most studies also represents a limitation that prevented us from isolating the effect of IF in some specific outcomes. Several studies on the interaction of IF and exercise performance did not report effect sizes or statistical power in their analyses. Hence, it is important to implement rigorous experimental designs in future studies to ensure that appropriate conclusions can be drawn. Results from this systematic review are also limited by a lack of studies on IF with a representative sample size. The high level of heterogeneity found between studies (e.g., different endpoints and time points for performance testing) could not be explored with moderator analyses due to the limited number of studies. Finally, due to the limited number of studies (<10 per outcome, with the exception of body mass), publication bias was not examined. The reader should be aware of these limitations when considering the practical/clinical influence of IF on physical performance and body composition.

Further research is needed to explore the interaction between IF and exercise performance in women. It is fundamental to ensure that both volume and intensity of training are well maintained during the experiments. Nutrient intake should also be closely monitored over time and it is mandatory to determine the role of altered sleep and dehydration on the relationship between IF and exercise performance. Changes in fat free mass should also be closely monitored. More research is also warranted to explore the interaction between IF and changes in muscle strength, using designs that match protein intake at optimal levels for muscular hypertrophy. Finally, more studies related with the effects of IF on performance are also required to unravel the physiological mechanisms and energy pathways that allow athletes to maintain or even improve performance during IF.

## 5. Conclusions

Overall, the existent research examining the effects of IF on physical parameters and body composition has provided conflicting data. Our meta-analytic findings reveal significant pooled estimate effects of IF on VO_2max_. Additionally, they suggest that IF may also have some positive effects on decreasing body mass and fat mass. For muscle strength and anaerobic capacity, the pooled estimate effects of IF were largely non-significant. Thus, the current review indicates that, while Ramadan IF may lead to impairments in aerobic capacity, TRF may be effective for improving it. All authors have read and agreed to the published version of the manuscript.

## Figures and Tables

**Table 1:** Summary of studies evaluating the effects of intermittent fasting on physical performance and body composition.

Reference	Parameters	Participants	Age	Trial Length & Intervention	Aerobic Changes	Anaerobic Changes	Strength Changes	Body Composition Changes (*Secondary Outcome*)
[34]	BC; VO_2max_; V_E_; VCO_2_; R; HR; Blood sample.	6 physically active men (A). 7 sedentary men (S).	Group A, 35.5 ± 1.6 years Group S, 37.6 ± 2.3 years	1 week before RF. 2 weeks after the start of RF. 4th week of RF.	↓ HR (submax exercise) in S group during Ramadan. Significant ↓ of HR (submax exercise) in A group during Ramadan.	-	-	Total BM and FM ↓ non-significantly in both groups.
[4]	Anaerobic power; Anaerobic capacity; HR; BC; Blood lactate.	10 male elite power athletes	22.3 ± 1.3 years	3 days before Ramadan (pre-RF). The last 3 days of Ramadan (end-RF). The last 3 d of the 4th week after the end of Ramadan (post-RF).	-	No adverse effects on anaerobic power and capacity.	-	No significant differences in body weight, BM index, FFM, % of body fat (↑ 0.2%).
[35]	Pmax; Pmean; Ppeak; BC.	12 healthy males	22.6 ± 1.3 years	1 week before Ramadan. 2nd week of Ramadan. 4th week of Ramadan. 2 weeks after Ramadan.	-	Negative effect of Ramadan on anaerobic performance during the afternoon and evening. No effect on anaerobic performance in the morning.	-	No significant changes for body mass.
[36]	Speed; Power; Agility; Endurance; Passing and dribbling skills	85 junior soccer players	18.0 years	3 weeks before Ramadan. 2nd week of Ramadan. 4th week of Ramadan. 3 weeks after Ramadan.	No change in fasted 10 or 30 m run during the afternoon, but ↑ fatigue index during Ramadan.	-	↑ in vertical jump height from pre-Ramadan to the 2nd and 4th week of Ramadan, especially in the afternoon. Significant ↑ in vertical jump performance from pre-to post-Ramadan, especially in the afternoon.	-
[10]	Mean speed; Mean HR; Distance covered in 30 min TT run; Physiological variables taken before and after the 60 min exercise runs.	10 moderately trained male runners	27.3 ± 7.2 years	1 week before Ramadan. During Ramadan. 1 week after Ramadan.	Shorter distance covered during the 30 min TT run during Ramadan.	-	-	-
[17]	VO_2max_; Submaximal VO_2_ kinetics; Anthropometric parameters; MVC; Running efficiency; Running Performance	18 well-trained Muslim runners (EG, *n* = 9; CG, *n* = 9)	23.6 ± 2.9 years	1 week before Ramadan. Last week of the fasting period. After Ramadan.	Significant ↓ in running speed at VO_2max_ in the EG. No changes in VO_2max_ or running efficiency. Significant improvement of VO_2_ kinetics in EG. Significant ↓ in 5000 m running in EG.	-	No significant difference was observed in MVC between groups. Significant ↓ in MVC was observed, after Ramadan, only in the EG.	No significant differences on BM and FM between groups before and after (EG ↓ 0.8% FM; CG ↓ 1.3% FM).
[21]	PA; BC; Muscle force; EMG activity.	11 moderately active Muslim males	31.0 ± 3.0 years	1 month and 1 week before Ramadan. During Ramadan. 1 month after Ramadan.	-	-	Maximal force was maintained during Ramadan. Maximal isometric torque measurements were not significantly different between sessions for both the knee extensors and flexors.	Little influence of Ramadan on BM and BC.
[37]	Upper arm girth; Isometric strength; Blood sample; RPE; BMI.	29 participants (19 men, 12 women). Post-prandial group (*n* = 5 men, *n* = 5 women). Fasting group (*n* = 12 men, *n* = 7 women)	22.0 ± 3.3 years	Pre-fasting. Post-fasting - session #1, 2, 3, 4 and 5. (8 h water-only fast)	-	-	Upper arm girth ↑ significantly within each group. Elbow extension and isometric strength ↓ significantly over time within each group. Less induction of muscle damage (EG).	BMI was similar between groups and sessions.
[38]	Wmax-A; Wmax-L; VJH; HGF; Anthropometric data; Hemoglobin and hematocrit	20 trained men (RF, *n* = 10). (CG, *n* = 10)	RF, 21.8 ± 1.9 years; CG, 20.5 ± 1.0 years	Before Ramadan. End of the 1st week of Ramadan (R1). During the 4th week of Ramadan (R4).	-	-	Significant ↓ in the Wmax-A and Wmax-L during Ramadan. No significant change in VJH and HGF during Ramadan.	BM and BM index was ↓ at R1 and R4. FM was lower at R4 (↓0.3%).e
[39]	VO_2max_; MAP; Reaction time; Anthropometric variables.	10 adolescents karate athletes	18.5 ± 0.5 years	3 weeks before Ramadan. At the end of the first week of Ramadan. During the 4th week of Ramadan. 3 weeks after Ramadan.	No significant changes at VO_2max_, MAP and HR_max_ during Ramadan. No effect on reaction time.	-	-	No significant changes in BM and FM during Ramadan.
[9]	VO_2max_; HIE; Flexibility; Agility; Vertical jump; Handgrip strength.	77 untrained Muslim men (EG, *n* = 37; CG, *n* = 40)	EG, 22.6 ± 1.8 years. CG, 23.0 ±1.5 years	1 week pre-Remadan. During Ramadan. 2 weeks post-Ramadan.	Significant ↓ in VO_2max_.	Significant ↓ in HIE	Vertical jump did not show any alteration. Non-significant variation in handgrip strength.	↓ BM, but not significant.
[40]	Ventilatory variables; Metabolic variables.	16 healthy and active men (FAST, *n* = 8) (FED, *n* = 8)	FAST, 20.9 ± 2.0 years; FED, 21.3 ± 2.5 years	3 d/week for 6 weeks in the fasted state (Night fast)	Significant ↑ of VO_2max_ for both groups. ↓of time to run 3000 m (EG and CG).	-	-	Fat oxidation was not modified by training.
[6]	TRF; ND; BC; Strength; Blood sample.	34 resistance-trained males (TRF, *n* = 17; ND, *n* = 17)	TRF, 29.2 ± 3.8 years; ND, 28.5 ± 3.5 years	Tested 8 weeks pre-RT. 8 weeks of TRF. Tested 8 weeks post-RT. (Intermittent fast 8/16 h)	-	-	Maintained in TRF and ND.	Significant ↓ FM TRF (−16.4%); FFM was maintained in TRF and ND.
[41]	Maximal speed; Mean speed; Maximal power; Mean power; Vertical stiffness; Vertical COM; FI; Blood sample; HR; Body mass; RPE;	21 active males	29.8 ± 5.9 years	Fed/control session (pre-3 d of IF). 3 d of IF.	-	Significant effect of fasting on maximal and mean speed, maximal and mean power, vertical stiffness and vertical COM displacement. Sprint speed and mean power significantly ↓ from the CG. 3 d of IF impaired speed and power through a ↓ in vertical stiffness.	-	No significant changes on BM loss.
[2]	RT-TRF; BC; Muscle strength.	18 active men (RT-TRF, *n*= 10; RT-ND, *n* = 8)	RT-ND, 22.0 ± 2.4 years. RT-TRF, 22.9 ± 4.1 years	8 weeks of RT-ND. 8 weeks of RT-TRF. TRF for 4 d/week. Analysis 4 and 8 weeks post study. (No fasting on training days; intermittent fasting on non-training days 4/20 h)	-	-	↑in upper and lower body muscle strength in ND and TRF groups, but ↑ were better in RT-TRF group. No changes after 4 weeks.	TRF-RT group lost up to 5.5% and 22% of initial BM and FM, respectively.
[42]	VO_2max_; HR_max_; MVIC; BC; Sleep; Tre; Tsk; RPE; Blood markers; Training load.	14 male trained Muslim football players	21.8 ± 2.4 years	Before Ramadan. During Ramadan. After Ramadan.	mLIST were significantly faster in both CG (before and after Ramadan) as compared to Ramadan fasting group. An adverse effect on prolonged performance during Ramadan fasting.	-	Minimal ↓ in fatigue within the working muscles and in the neuromuscular activation. No changes were verifying in MVIC of the upper limb. No significant differences on knee extension MVIC over trials in each group.	BM at pre- and post-exercise was not significantly different before, during and after Ramadan.
[43]	VO_2peak_; Anaerobic power; BC; Blood sample; Urine sample.	20 healthy male	EG, 21.0 ± 1.0 years; CG, 20.0 ± 1.0 years	7 d of DS and EF. Baseline data (day 0). Day #2, 4, 6, 8, 10 (exercise test). (Intermittent fasting with lunch deprivation)	-	↓ WnT power in EG during Day 2, Performance returned to baseline results from Day 4. HIT TTE in EG ↓throughout the 10-day period, but seems to recovered at the end of the experiment.	-	↓ BM in the EG (−1.55%).
[11]	MVIC; Voluntary activation level; Neuromuscular efficiency Body mass.	10 healthy male	22.1 ± 2.0 years	Before Ramadan. During Ramadan. After Ramadan.	-	-	MVIC↓during Ramadan. Voluntary activation level↓during Ramadan. Neuromuscular efficiency remained unaffected by Ramadan.	BM remained unaffected by Ramadan.
[44]	VO_2_ peak, PO, PPO, Mean PO, FI, Blood samples, VT1, VT2	20 male cyclists (SIT FAST, *n* = 11; SIT CHO, *n* = 9).	SIT FAST, 33.3 ± 7.2 years SIT CHO 34.0 ± 8.2 years	SIT in overnight fasted state or with carbohydrate supplementation. Groups performed 3 times per week for 4 weeks.	PPO was significantly ↑ in SIT CHO compared to SIT FAST. No differences in post-training VO2 VT1 nor VT2 between groups.	-	-	-
[45]	VO_2_max; VCO_2_; HR; BM; Time to exhaustion; RER; Fat and CHO oxidation rate; Muscle and blood samples.	20 young male. (Fasted state, *n* = 10; Fed state CHO, *n* = 10)	d.s	6 weeks endurance training program, 4 days per week, in fasted or ingested CHO before and during training sessions.	VO_2_ max and time to exhaustion, between fasted and fed groups, were similar in the pre-test and ↑ 9% after training, but not significantly.	-	-	↑ fat mass oxidation in fast group (+ 21%), but not significant. Body weight ↓ in both groups (not significantly).
[46]	VO_2_ peak; VO_2_; VCO_2_; RER; BM; Muscle and blood samples.	20 moderately active males. (Fast, *n* = 10; CHO, *n* = 10)	21.2 ± 0.4 years	Endurance training program, 6 weeks of 3 days per week, in fasted or CHO fed state.	VO_2_ peak ↑ within group but was not different between groups.	-	-	No significant changes on body mass loss.
[47]	VO_2_ peak; VO_2_; VCO_2_; RER; HR; Muscle and blood samples.	8 males	25 ± 2 years	Cycling in fasted state or 2 h following ingestion of CHO.	VCO_2_ ↓ significantly during fast exercise. VO_2_; HR and RER were not different during exercise between trials.	-	-	-
[48]	VO_2_ max; VO_2_ peak; biochemical analyses.	8 females untrained 6 males untrained	26.6 ± 5.8 years	4 weeks of 5 days per week endurance cycle ergometer in overnight-fasted or acutely fed state.	Fast group showed ↑ in VO_2_ max than Fed group. Peak power ↑ more in fasted group compared to CG.	-	-	-
[49]	HR; Blood samples; Reaction time; cognitive performance.	21 physically active, healthy Muslims males.	29.8 ± 5.9 years	3 days of IF	-	Simple and complex reaction times ↑ during the 3d-IF after 2 bouts of intensive RS.	-	-
[24]	Skeletal muscle hypertrophy; muscular performance; BM; FFM; Commonly physiological and metabolic variables.	40 resistance trained females. (CG, *n* = 14; TRF, *n* = 13; TRF_HMB_, n13).	CG, 22.0 ±2.4 TRF, 22.1 ± 2.1 TRF_HMB_, 22.3 3.4	8 weeks of supervised RT in TRF state with or without HMB.	-	-	TRF did not attenuate FFM, muscle hypertrophy, or developments in muscle performance.	FFM ↑ in three groups without differences between all. Significant ↓ in FM in TRF group.
[50]	PA level; Anthropometric status; Body composition; Dietary information.	33 healthy young males	21.85 ± 1.87 years	Before Ramadan; 1st week of Ramadan; Last week of Ramadan.	Statistically significant ↓ in high intensity PA in 1st w of RF. Statistically significant ↓ in moderate PA in 1st w of RF compared with before RF.	-	-	Significant ↓ in BM and %FM at last week of RF compared to before RF.
[51]	CMJ; RS test; YYIRT1	30 amateur soccer players. (MTG, *n* = 10; ETG, *n* = 10; CG, *n* = 10)	22.9 ± 1.3 years	Before Ramadan. After Ramadan.	-	After RF YYIRT1 ↑in the morning and in the evening. ↑in RS training (fasted state) in the morning or in the evening. But significant difference compared to before and after Ramadan in the morning.	No differences were observed in CMJ before and after RF for any group.	-
[52]	Blood samples; VO_2_ max; HR; BM; Endurance exercise performance; metabolic measurements; Mood state and daytime sleepiness.	12 Muslims men runners.	27.9 ± 7.2 years.	Endurance running performance during Ramadan, after ingesting LGI or normal mixed CHO food as *sahur* meal.	TT distance ran was statistically significant ↓ in LGI vs. CG.	-	-	No significant changes in BM.

Abbreviations. BC, body composition; BIA, bioelectrical impedance analyser; BM, body mass; CG, control group; CHO, carbohydrate; CMO, vertical center of mass; DS, dietary standardization; d.s, did not say; EF, exercise familiarization; EG, experimental group; EMG, electromyography; ETG, evening training group; FFM, fat free mass; FI, fatigue index; FM, fat mass; HGF, handgrip force; HIE, high-intensity effort; HIT, high-intensity cycling test; HMB, β-hydroxy β-methylbutyrate supplementation; HR, heart rate; IPAQ, international physical activity level questionnaire; LGI, low glycemic index; MAP, maximal aerobic power; MAS, maximal aerobic speed; mLIST, Loughborough intermittent shuttle test; MTG, morning training group; MVC, maximal voluntary contraction; MVIC, maximal voluntary isometric contraction; ND, normal diet; PA, physical activity; Pmax, maximal power; Pmean, mean power; PO, power output; Ppeak, peak power; PPO, Peak power output; R, respiratory exchange ratio; RF, Ramadan fasting; RPE, rating of perceived exertion; RS, Repeated sprints, RT, resistance training; RT-ND, resistance training and normal diet; RT-TRF, resistance training with time-restricted feeding; SIT, sprint interval training; Tre, rectal temperature; TRF, time-restricted feeding; Tsk, skin temperature; TT, time trial; TTE, time-to-exhaustion; VE, ventilation; VCO2, carbon dioxide output; VJH, vertical jump height; VO2max, maximal oxygen consumption; VO2peak, peak oxygen consumption; VO2, oxygen consumption; VT1, first ventilatory threshold; VT2, second ventilatory threshold; Wmax-A, maximal anaerobic power of the arms; Wmax-L, maximal anaerobic power of the legs; WnT, Wingate test; YYIRT1, yo-yo intermittent recovery test level 1.

**Table 2:** Quality assessment of all included studies.

Reference	Selection Bias	Study Design	Confounders	Data Collection Method	Withdrawals	Global
[34]	1	1	3	3	3	Weak
[4]	1	1	3	3	3	Weak
[35]	1	1	3	3	3	Weak
[36]	2	1	3	3	3	Moderate
[46]	1	2	3	3	3	Moderate
[10]	1	1	3	3	3	Weak
[48]	1	2	3	3	3	Moderate
[45]	1	2	3	3	3	Moderate
[17]	1	1	3	3	3	Weak
[21]	1	1	3	3	3	Weak
[37]	1	1	3	3	3	Weak
[38]	1	1	3	3	3	Weak
[52]	3	2	3	3	3	Strong
[39]	1	1	3	3	3	Weak
[9]	2	1	3	3	3	Moderate
[40]	1	3	3	3	3	Moderate
[41]	1	2	3	3	3	Moderate
[6]	1	3	3	3	3	Moderate
[2]	1	3	3	3	3	Moderate
[49]	3	2	3	3	3	Strong
[42]	1	3	3	3	3	Moderate
[51]	2	2	3	3	3	Strong
[43]	1	3	3	3	3	Moderate
[44]	2	2	3	3	3	Moderate
[11]	1	1	3	3	3	Weak
[50]	1	1	3	3	3	Weak
[24]	2	3	3	3	3	Strong
[47]	1	2	3	3	3	Moderate

Note. 1, weak; 2, moderate; 3, strong.

**Table 3:** Meta-analytic results for the effects of intermittent fasting on physical performance.

					Heterogeneity		
Primary Outcomes	Point Estimate	CI Lower	CI Upper	*p*-Value	Q-Value	*p*-Value	I-Squared
**Muscle strength**							
Vertical Jump (cm) *							
All studies							
Only Ramadan IF studies	0.012	−0.324	0.348	0.945	43.598	<0.001	95.413
Only TRF studies							
Vertical Jump (cm) **							
All studies							
Only Ramadan IF studies	0.021	−0.316	0.358	0.902	1.23	0.541	<0.001
Only TRF studies							
**Aerobic capacity**							
VO_2_max (L·min^−1^) *							
All studies	0.051	−0.275	0.376	0.761	1.205	0.877	<0.001
Only Ramadan IF studies	−0.046	−0.417	0.324	0.806	0.055	0.973	<0.001
Only TRF studies	0.375	−0.303	1.053	0.278	0.007	0.932	<0.001
VO_2_max (L·min^−1^) **							
All studies	−1.045	−1.488	−0.602	**<0.001**	80.05	<0.001	96.252
Only Ramadan IF studies	−2.204	−2.745	−1.663	**<0.001**	21.229	<0.001	95.289
Only TRF studies	1.315	0.543	2.087	**0.001**	5.323	0.021	81.212
30 min distance (m) **							
All studies							
Only Ramadan IF studies ***	0.187	−0.405	0.78	0.536	0.044	0.835	<0.001
Only TRF studies							
**Anaerobic capacity**							
Mean power (w) *							
All studies	0.04	−0.753	0.834	0.921	123.219	<0.001	98.377
Only Ramadan IF studies	0.355	−0.442	1.151	0.383	38.813	<0.001	97.424
Only TRF studies							
Peak Power (w) *							
All studies	0.758	−0.019	1.535	0.056	113.389	<0.001	98.236
Only Ramadan IF studies	−0.11	−0.912	0.691	0.788	40.109	<0.001	97.507
Only TRF studies							

**Note.** * Pre-post tests, IF intervention; ** Pre-post tests, IF intervention vs. control; *** Comparison of means between the Ramadan IF interventions and the control groups; * Vertical jump height, Ramadan IF studies (k = 3); ** Vertical jump height, Ramadan IF studies (k = 3); * VO_2max,_ all studies (k = 5); * VO_2max,_ Ramadan IF studies(K = 3); * VO_2max,_ TRF studies (K = 2); ** VO_2max,_ all studies (k = 4); ** VO_2max,_ Ramadan IF studies(K = 2); ** VO_2max,_ TRF studies (K = 2); * WnT mean power output, all studies (k = 3); * WnT mean power output, Ramadan IF studies (k = 2); * WnT peak power output, all studies (k = 3); * WnT peak power output, Ramadan IF studies (k = 2); and *** 30 min distance, Ramadan IF studies (K = 2). Abbreviations. CI, confidence interval; Ramadan IF, Ramadan intermittent fasting; TRF, time-restricted feeding; VO_2max_, maximum oxygen consumption; WnT, Wingate.

**Table 4:** Meta-analytic results for the effects of intermittent fasting on body composition.

					Heterogeneity		
Secondary Outcomes	Point Estimate	CI Lower	CI Upper	*p*-Value	Q-Value	*p*-Value	I-Squared
Body Mass (Kg) *							
All studies	−0.435	−0.811	−0.059	**0.023**	30.718	0.001	64.19
Only Ramadan IF studies	−0.638	−1.248	−0.029	**0.04**	23.256	0.001	74.2
Only TRF studies	−0.152	−0.476	0.173	0.359	2.635	0.621	<0.001
Body Mass (Kg) **							
All studies	−0.058	−0.388	0.272	0.732	3.58	0.611	<0.001
Only Ramadan IF studies	−0.313	−0.955	0.329	0.339	0.508	0.476	<0.001
Only TRF studies	0.034	−0.351	0.419	0.862	2.246	0.523	<0.001
Fat Mass (%) *							
All studies	−0.848	−1.193	−0.502	**<0.001**	7.307	0.121	45.256
Only Ramadan IF studies	−0.910	−1.284	−0.535	**<0.001**	6.616	0.085	54.655
Only TRF studies							
Fat Mass (%) **							
All studies	−0.224	−0.751	0.302	0.404	0.029	0.986	<0.001
Only Ramadan IF studies	−0.215	−0.853	0.423	0.508	0.026	0.872	<0.001
Only TRF studies							

**Note.** * Pre-post tests, IF intervention; ** Pre-post tests, IF intervention vs. control; * Body mass, all studies (k = 12); * body mass, Ramadan IF studies (k = 7); * body mass, TRF studies (k = 5); ** body mass, all studies (k = 6); ** body mass, Ramadan IF studies (k = 2); ** body mass, TRF studies (k = 4); * relative fat mass, all studies (k = 5); * relative fat mass, Ramadan IF studies (k = 4); ** relative fat mass, all studies (k = 3); and ** relative fat mass, Ramadan IF studies (k = 2). Abbreviations. CI, confidence interval; Ramadan IF, Ramadan intermittent fasting; TRF, Time-restricted feeding.

## Abbreviations

IF	Intermittent fasting
Ramadan IF	Ramadan intermittent fasting
TRF	Time-restricted feeding
SMD	Standardized mean difference
VO_2max_	Maximum oxygen uptake
CR	Caloric restriction
PRISMA	Preferred reporting items for systematic reviews and meta-analysis
EPHPP	Effective public health practice project
CMA	Comprehensive meta-analysis
CI	Confidence interval
